# Strategy and technology to prevent hospital-acquired infections: Lessons from SARS, Ebola, and MERS in Asia and West Africa

**DOI:** 10.1186/s40779-017-0142-5

**Published:** 2017-10-27

**Authors:** Sanjeewa Jayachandra Rajakaruna, Wen-Bin Liu, Yi-Bo Ding, Guang-Wen Cao

**Affiliations:** Department of Epidemiology, Second Military Medical University, Shanghai, 200433 China

**Keywords:** SARS, Ebola, MERS, Infection control, Hospital-acquired infections, Strategy, Technology

## Abstract

Hospital-acquired infections (HAIs) are serious problems for healthcare systems, especially in developing countries where public health infrastructure and technology for infection preventions remain undeveloped. Here, we characterized how strategy and technology could be mobilized to improve the effectiveness of infection prevention and control in hospitals during the outbreaks of Ebola, Middle East respiratory syndrome (MERS), and severe acute respiratory syndrome (SARS) in Asia and West Africa. Published literature on the hospital-borne outbreaks of SARS, Ebola, and MERS in Asia and West Africa was comprehensively reviewed. The results showed that healthcare systems and hospital management in affected healthcare facilities had poor strategies and inadequate technologies and human resources for the prevention and control of HAIs, which led to increased morbidity, mortality, and unnecessary costs. We recommend that governments worldwide enforce disaster risk management, even when no outbreaks are imminent. Quarantine and ventilation functions should be taken into consideration in architectural design of hospitals and healthcare facilities. We also recommend that health authorities invest in training healthcare workers for disease outbreak response, as their preparedness is essential to reducing disaster risk.

## Background

Hospital-acquired infections (HAIs) are serious public health problems that increase the morbidity, mortality, and avoidable healthcare cost worldwide. Millions of people suffer from HAIs, ranging from the common cold to severe infections, annually. Data from 66 hospitals in 23 countries show that the prevalence rate of HAIs is 7.1% in Europe, and this number is doubled or more in developing countries, such as Uganda [1, 2]. Furthermore, HAIs are partly responsible for the repeated outbreaks of several fatal infectious diseases in Asia and West Africa, such as severe acute respiratory syndrome (SARS), Middle East respiratory syndrome (MERS), and Ebola [3, 4]. Hospital-borne outbreaks of these diseases reflect the vulnerability of the affected hospitals in low- and middle-income countries (LMICs) [5]. For example, at the early stage of the Ebola outbreak in 2013, the human resource policy of public health in Guinea was blamed for failing to provide adequate manpower for research, information system, and transportation of medical supplies [6]. The lack of personal protective equipment (PPE), a common problem in most hospitals in West African counties affected by Ebola, increased the exposure risk of health care workers (HCWs). A scientific and effective emergency plan is important for the early response and comprehensive interventions of HAI outbreaks [7]. The World Health Organization (WHO) suggests that all countries should have a plan in place, even if the risk of highly contagious condition seems remote. The lack of proper communication contributes to the spread of diseases. Effective public health response plans could reduce fatalities during outbreaks of SARS and MERS in Asia [8]. In addition to the policies, emergency plans, and equipment, education and training are also very important for the prevention and control of HAIs, especially for those caused by unusual, novel, or fatal pathogens. The data and knowledge of these pathogens should be communicated effectively from laboratories to leaders, managers, and HCWs to improve the response plan and diagnostic capabilities [9].

Recent outbreaks of SARS, Ebola, and MERS became serious public health events. SARS is caused by a new type of coronavirus, termed SARS-CoV, that may have evolved from bat coronaviruses [10]. SARS causes fever, cough, serious lung problems, and even death. In 2002—2003, SARS outbreaks affected China and other Asian countries, and then spread to Canada [11, 12]. Worldwide, 20% of the laboratory-confirmed SARS patients were HCWs, who had a fatality rate of 40% [13]. Ebola is an acute viral hemorrhagic disease endemic to West Africa [14–16]. People can become infected with Ebola through direct contact with the sweat, urine, blood, or stool of an Ebola-infected individual. By the end of March 2016, a total of 28,646 Ebola cases and 11,323 related deaths were reported [17]. In particular, Guinea, Liberia, and Sierra Leone were heavily affected. HAIs of Ebola is one of the main barriers to reducing Ebola in West Africa [17]. MERS is caused by a novel coronavirus, MERS-CoV, and was first reported in Saudi Arabia in 2012 [18]. Movement of zoonotic reservoirs and consumption of the meat from infected animals aggravate the transmission of MERS-CoV. Nosocomial transmission was frequently reported [19]. In the Arabian Peninsula, the fatality rate of MERS reached 30% [20, 21]. In this paper, we reviewed studies on HAIs of these 3 fatal infectious diseases. Key issues of strategy, technology, and human factors that affect HAIs were summarized to improve the capability of HAIs prevention and hospital vulnerability assessment, especially in LMICs.

## Research status of HAIs prevention in West Africa and Asia

Scientific publications on SARS, Ebola, and MERS were reviewed with the task of identifying the key strategic and technological issues that influence HAIs. We searched the PubMed, WHO, and American Centers for Disease Control and Prevention (CDC) databases using Medical Subject Heading (MeSH) terms “hospital infection”, “health care associated infection”, “Ebola”, “Middle East respiratory syndrome”, and “severe acute respiratory syndrome”, as well as the individual corresponding free terms. A total of 868 articles were found. Of those, 795 studies were excluded for one or more of the following reasons: 1) not published as a full report; 2) no reporting of HAIs in Asia or West Africa; 3) no primary reporting of HAIs with Ebola, MERS, or SARS. Of 73 articles, 24 were selected because the risk factors of HAIs were thoroughly analyzed. Of these 24 HAIs studies, 12 reported SARS in China, Singapore, and Vietnam; 6 reported Ebola in West Africa; and 6 reported MERS in South Korea and Saudi Arabia.

Based on these 24 studies, strategy, technology, and human factors were identified as the major variables for HAIs prevention. The primary issues of these 3 variables are presented in Table 1. Strategy issues were found to reflect a hospital’s vulnerability to HAIs at the management level. The main task of prevention strategy for HAIs is to provide resources and adequate health workers to respond to any form of disaster, including epidemics [22]. The technology factors, including vehicles, public health infrastructure, transportation, communication, and other hospital equipment, were found to be important for an effective response to disease outbreaks. Human resources was found to be one of the major factors for preventing HAIs. Furthermore, other shortfalls that led to poor HAIs prevention in West Africa and Asia were also summarized.

**Table 1:** Major issues for HAIs prevention

Item	Issue
Strategy	Disaster response plan; Communication plan; Funding; Training; Availability of personal protective equipment; Presence of case definitions; Standard operating procedures and guidelines; Timely decision-making
Technology	Hospital design; Adequate lighting; Adequate ventilation; Availability of cleaning equipment; Availability and state of laboratory equipment; Computers; Internet and communication facilities; Transport system such as utility cars and ambulances; Functional online appointments systems and electronic payment system for reducing patient density in the hospital’s OPD
Human factor	Knowledge; Prevention condition; Attitudes; Use of personal protective equipment; Hand washing; Handling of patient’s excreta and waste; Barrier nursing and isolation; Timely reaction to suspected cases; Timely reporting; Alarm raising and response to disaster call out

*OPD* Outpatient department

### Strategy factors

Healthcare strategy is a key driver of successful infection control in healthcare settings. Policies and plans of healthcare service, patient delivery, human resources, financial and material resources, and communication are all important for the improvement of the management strategy of a hospital [23]. A strategy for the prevention of HAIs should not only provide bio-security of HCWs and patients but also ensure a positive practice environment. For example, many medical instruments can become sources of infection because of the difficulty in cleaning them [24]. However, emergency plans are necessary for healthcare systems to respond effectively to an outbreak of a highly infectious disease [25]. Studies in North America show that nearly all hospitals (99.0%) have emergency medical response plans for chemical emergencies, natural disasters, disease outbreaks, and biological attacks [26]. These plans include HCWs training, patient evacuation, communication, and medical resource supporting. Furthermore, emergency medical response protocols should be updated and distributed in time, thus minimizing confusion from healthcare staff when the disaster strikes [27].

**Fig. 1:**
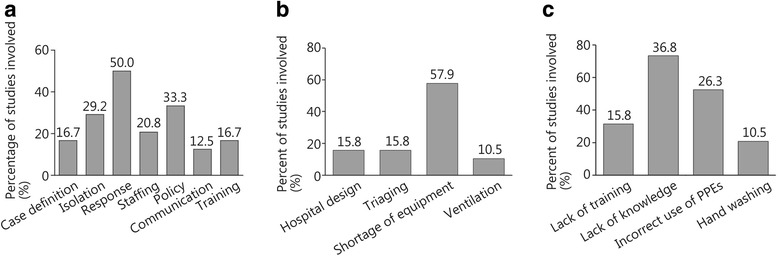
Percent of studies that report the shortfalls which impact HAI prevention and control. **a** Strategy shortfalls; **b** Technology shortfalls; **c** Human factors

According to the related studies, 7 strategy shortfalls related to the failure of HAIs prevention and related countries are summarized (Table 2). The major challenge observed was delayed response to the outbreaks, which was reported by 50.0% HAIs studies of Ebola, SARS, and MERS in Asia and West Africa. In countries of the Mano River Union (Guinea, Sierra Leone, and Liberia), almost all HAIs cases of Ebola were related to delayed response. Policy deficiency (33.3%) and lack of standard case definition (16.7%) were responsible for this international catastrophe [28]. Additionally, lack of isolation (29.2%) and poor training (16.7%) were found to have an impact on the spread of nosocomial infections. Poor communication was also found to be a major cause of disease outbreaks in West Africa and Asia (Fig. 1a) [18]. Timely communication among healthcare facilities, public organizations, and government was the key factor of emergency response [29]. To ensure the initial case and suspected outbreak can be identified in time, report procedures should be known by all members of a healthcare institution.

**Table 2:** Strategy shortfalls of HAIs prevention and related countries

Item	Country
Lack of case definition	Sierra Leone [38], Guinea [38], Liberia [38], China [39], Vietnam [3, 40], South Korea [3]
Lack of isolation	Sierra Leone [41], Guinea [41], Liberia [41], China [35, 42, 43], Vietnam [40], South Korea [41, 44], Singapore [45]
Delayed response	Sierra Leone [38, 41, 46], Guinea [38, 41, 46], Liberia [38, 41, 46, 47], China [39, 43, 48], Vietnam [3, 40], South Korea [41, 44, 49], Saudi Arabia [50]
Limited staffing	Sierra Leone [46], Guinea [46, 51, 52], Liberia [46, 51, 52], China [53, 54]
Policy deficiency	Sierra Leone [38, 47], Guinea [38], Liberia [38, 47], China [55, 56], Vietnam [40], South Korea [57], Singapore [45], Saudi Arabia [50]
Poor communication	Guinea [41], Liberia [41], China [58], South Korea [50], Saudi Arabia [50]
Poor training	Sierra Leone [46] Guinea [46], Liberia [46], China [42, 59], South Korea [57]

### Technology factors

Unsuitable isolation design, poor facility ventilation, ineffective triaging, and a shortage of equipment were the key technology shortfalls of many hospitals in Asia and West Africa, which led to the failure of HAIs prevention (Fig. 1b) [7, 9, 30–32]. Of the 24 publications, 57.9% reported that the equipment and machinery of many hospitals in Asia and West Africa failed to support the need of HAIs control. The most important equipment for HAIs prevention is PPE, which is composed of disposable masks, gloves, and isolation gowns. Unqualified equipment can increase the risk of HAIs. In Singapore, some HCWs reportedly contracted SARS due to loose-fitting N95 masks [33]. Facilities of communication, hand washing, and changing are also indispensable in controlling HAIs, especially in times of outbreaks. However, 15.8% and 10.5% studies reported that hospital designs and ventilation were not suitable for effective isolation. In addition, ineffective triaging was identified as the cause for the rapid spread of HAIs in 15.8% of studies.

Isolation is the major method of HAI control, but the method of implementation depends on hospital design. Most hospitals and healthcare facilities in Asia and West Africa were not designed to handle large numbers of patients with highly infectious diseases at short notice. Therefore, they had only a few spaces that could act as isolation wards [23]. Isolation wards away from other regions of hospital are crucial to prevent cross-infection [34]. A well-designed isolation area should have at least 3 regions: heavily contaminated, lowly contaminated, and non-contaminated. Isolation wards with a single bed are most suitable for preventing transmission through droplets [9]. In wards with two or more beds, bed distance should be more than 3 ft, and curtains are necessary. Another basic feature of isolation ward is the regulation of airflow, which can reduce the cross contamination of airborne pathogens. Architectural design of compartments including the locations of windows and doors can ensure the natural airflow. Exhaust ventilators of the isolation rooms should be located far from the intakes of other areas to avoid contamination. Ventilation facilities can improve bio-security by maintaining air pressure. For isolation wards of highly infectious diseases, environments with negative pressure are required. In contrast, positive pressure should be provided to operating rooms, intensive care unit (ICU), and other important places. The regulation of airflow is especially important for the prevention of pathogens such as SARS-CoV, which can not be filtered through a high-efficiency particulate air (HEPA) filter [9]. Overcrowding, a common problem of most hospitals in Asia and West Africa, increased the risk of HAIs, especially during the outbreak of diseases. Therefore, in addition to isolation, another important method of HAIs prevention is reducing the density of patients in outpatient departments [8]. Appointments and effective triaging can ease overcrowding. Guiding services and information desks can save time for patients by providing basic information, such as the locations of examination rooms, the dispensary, and the laboratory [8, 32]. Importantly, online systems of patient appointments and information queries can efficiently reduce the patient density of some areas of a hospital.

### Human factors

An effective healthcare system depends on the cooperation of all hospital staff, including doctors, nurses, laboratory personnel, ambulance paramedics, and many other HCWs. Knowledge, professional skills, and attitude of hospital staff were found to be important to the prevention of HAIs during the outbreaks of SARS, Ebola, and MERS in Asia and West Africa [34]. Figure 1c presents the human factors that impacted HAIs prevention and control. The most important human factor was found to be lack of knowledge, reported by 36.8% of the studies.

Lack of knowledge can cause delayed response to suspected cases, poor triaging, and incorrect use of PPE. HCWs are more easily infected by suspected patients at the early stage of an outbreak before most HCWs have the necessary knowledge about case definitions and transmission methods of the pathogen [35]. This highlights the importance of timely education and training of hospital staff. The safety of HCWs is based on the assumption that they have been trained to adopt appropriate behaviors in infectious and non-infectious conditions [34]. It is important to note that the lack of training is considered a significant contributor to the spread of SARS, Ebola, and MERS [6, 36, 37]. To some extent, this fact reveals that the healthcare systems in Asia and West Africa lack prior preparedness. Response activities to disease outbreaks should always be emphasized even if there is no imminent risk [29]. Studies reviewed herein show that HAIs of these 3 fatal diseases can be prevented by using basic prevention strategies, such as hand hygiene protocols. Many nurses and doctors were infected because of the lax attitude toward daily precautions and standard operating procedures. This lax attitude may also lead to a delayed reaction to the suspected cases. However, there are relationships among these human factors. Most HCWs that lack knowledge about infection sources and transmission routes of the Ebola virus, MERS coronavirus, and SARS coronavirus, usually pay no attention to the use of PPE and hand hygiene practices. Some health care facilities in LMICs ignored the importance of providing enough safe water and sanitations for hand washing and of providing timely training for HCWs who took care of Ebola, MERS, and SARS patients directly at the early stage of an outbreak. This might also be due to the lack of knowledge in HAIs prevention. Without special training, HCWs may miss important details and steps when using PPE or washing hands. For example, HCWs may forget to remove personal items (such as jewelry, watches, and pens) before putting on PPE, forget to put on a second pair of gloves over the cuff, forget to perform hand hygiene on gloved hands before taking off PPE, or forget to perform hand washing after dealing with contaminated laundry.

## Conclusions

Hospitals and healthcare facilities in LMICs of Asia and West Africa had poor strategies for the prevention and control of HAIs. This situation led to delayed reactions, serious cross contamination, increased the mortality, and financial loss during the outbreaks of SARS, Ebola, and MERS. Technology shortfalls primarily included poor infrastructure and inadequate facility ventilation, which failed to support isolation and other efforts for HAIs prevention. Human factors were also responsible for the HAIs of SARS, Ebola, and MERS. Lack of knowledge can lead to the failure of identifying suspected cases and induce a lax attitude toward infection control methods, such as hand washing and the proper use of PPE. Lack of knowledge is also related to poor triaging in outpatient departments and emergency treatment areas.

Governments, CDCs, and hospitals are recommended to create strategies for emergency responses to infectious outbreaks. Additionally, this strategy should be updated frequently. It is important to ensure that all HCWs understand the policies and standard operating procedures for preventing HAIs. Quarantine and ventilation function should be taken into consideration in the architectural design of hospitals and healthcare facilities. The use of online systems for information queries and appointments is recommended to reduce a hospital’s patient density. Medical authorities should increase their investment in training HCWs to improve knowledge, professional skills, and attitude of HAIs prevention.

## Abbreviations

CDC: Centers for Disease Control and Prevention
HAIs: Hospital-acquired infections
HCWs: Healthcare workers
HEPA: High efficiency particulate air
ICU: Intensive care unit
LMICs: Low- and middle-income countries
MERS: Middle East respiratory syndrome
MeSH: Medical subject heading
OPD: Outpatient department
PPE: Personal protective equipment
SARS: Severe acute respiratory syndrome
WHO: World Health Organization
